# A double encryption protection algorithm for stem cell bank privacy data based on improved AES and chaotic encryption technology

**DOI:** 10.1371/journal.pone.0293418

**Published:** 2023-10-25

**Authors:** Li Wang, Xinyi Wei, Yuan Zhang, Yuan Gao, Qunfeng Niu

**Affiliations:** 1 School of Electrical Engineering, Henan University of Technology, Zhengzhou, Asia, China; 2 School of Information Science and Engineering, Henan University of Technology, Zhengzhou, Asia, China; 3 Henan Zhengda Stem Cell Bank Technology Company Limited, Zhengzhou, Asia, China; Al-Balqa Applied University Prince Abdullah bin Ghazi Faculty of Information Technology, JORDAN

## Abstract

The unique infinite self-renewal ability and multidirectional differentiation potential of stem cells provide a strong support for the clinical treatment. In light of the growing demands for stem cell storage, how to ensure personal privacy security and comply with strict ethical supervision requirements is particularly important. In order to solve the problem of low security of traditional encryption algorithm, we proposed a double encryption protection (DEP) algorithm for stem cell bank privacy data based on improved AES and chaotic encryption technology. Firstly, we presented the hash value key decomposition algorithm, through the hash value dynamic coding, cyclic shift, conversion calculation to get the key of each subsystem in the built algorithm. Secondly, DEP algorithm for privacy data is realized with two level of encryption. The first level of encryption protection algorithm used AES as the main framework, adding dynamic coding and byte filling based on DNA coding, and carries out dynamic shift of rows and simplified mixing of columns. The second level of encryption protection algorithm conducted random encoding, operation, diffusion and decoding based on the results of our proposed sequence conversion algorithm. Finally, we raised two evaluation indexes, the number of characters change rate (NCCR) and the unified average change intensity of text (UACIT) to measure the sensitivity of encryption algorithms to changes in plain information. The experimental results of using DEP shown that the average values of histogram variance, information entropy, NCCR and UACIT are116.7883, 7.6688, 32.52% and 99.67%, respectively. DEP algorithm has a large key space, high key sensitivity, and enables dynamic encryption of private data in stem cell bank. The encryption scheme provided in this study ensures the security of the private information of stem cell bank in private cloud environment, and also provides a new method for the encryption of similar high confidentiality data.

## 1. Introduction

Regenerative medicine has become a hot spot and frontier in the field of life science, providing new solutions for the treatment of many diseases [1, 2]. The stem cells, as the foundation of regenerative medicine, have great potential to improve human health. As an important resource of biomaterials for basic and translational stem cell research, stem cell banks have been rapidly expanding around the world [3]. According to a data from Coherent Market Insights, the global cell cryopreservation market will be worth $8.659 billion by 2022, and the CAGR is expected to reach 22.4% in 2022 to 2030 [4]. China has also established about 100 stem cell banks [5]. The stem cell bank is not only a repository of cells, but also a huge database that contains unique marks and records for the collection, processing, storage, transportation and management of each stem cell sample. In an era when open, shared, and affordable gene detection technologies are increasingly commonplace, the use of human biomaterials for cell research and intervention have raised ongoing concerns about protecting gene privacy [6]. All the data in the stem cell bank are personal privacy information, which have hidden dangers such as data leakage, tampering and counterfeiting in information sharing [7, 8]. If the information is obtained by attackers, it will cause great security risks [9]. In an effort to better achieve the interconnection between the private data of stem cell samples storage, handling, and other links in the private cloud environment and the blockchain platform. It is extremely important to ensure the security of the private data of stem cell bank through various encryption methods and comply with strict ethical regulatory requirements.

In the current research surrounding stem cell bank related fields, the human pluripotent stem cell registry (hPSCreg) has established a freely accessible cell line database to facilitate data sharing of cell characteristics with other platforms and cell banks around the world [10]. In [11], the unified management plan of the transplant registry unified management program (TRUMP) was developed in the purpose of promoting the unification and computerization of the hematopoietic stem cell transplantation registry, in view of the inconvenient query of the traditional paper registry. In [12], the authors established a basic data set of stem cell lines consisting of 33 data fields to improve the quality of cell line data and its availability in translational studies, in response to the unsatisfactory capture of specific cell line data. In [13], the integrated collection of stem cell bank data (ICSCB) is displayed to solve the problem of lack of standardized format of stem cell line data. It helps users to collect cell line information for multiple diseases and provider the latest and accurate cell line information. However, these studies around cells have focused on collecting cell data and establishing a standard cell line data format. With the rapid growth of the number of stem cell banks, more and more private data is stored in the information system. It is an extremely important link to study the encryption of private data in the information system of stem cell bank, which ensure the secure storage and transmission of private data. At present, there is no detailed explanation about how to store private information safely in the research of stem cell bank information system, especially less research on the encryption method of private data of stem cell bank.

Encryption algorithms based on the type of key used can be divided into symmetric and asymmetric encryption algorithm [14, 15]. The commonly used symmetric encryption methods include DES and AES [16, 17]. Compared with the asymmetric encryption system represented by RSA [18], the symmetric encryption system is simple and efficient [19]. DES algorithm cannot resist computer brute force cracking because the key length is only 56 bits [20]. AES encryption can choose key length according to the required level of encryption strength [21]. The AES algorithm is known for its high security and ease of implementation, and it has become the most widely used encryption algorithm in many security applications [22, 23]. In [24], a unified algorithm based on AES is proposed, which improves the shift rows and key expansion modules of AES algorithm, and adds a flip module. It provides the same encryption strength as AES while saving hardware resources. In [25], an enhanced AES algorithm is raised to improve the avalanche effect by modifying the substitution bytes and shift rows processes in the AES algorithm. In [26], a dynamic AES cryptosystem based on memristive neural network is projected, which realizes the dynamic encryption of ’one-time-one-secret’ and provides a larger key space. Although these encryption algorithms can ensure the security of plaintext information to a certain extent, they are greatly affected by the length of plaintext information and cannot meet the requirements of high confidentiality and tight ethical supervision in the storage and transmission of privacy data in stem cell bank.

Adleman proposed the method of DNA calculation for the first time, showing the advantages of high parallelism, fast computing speed and low energy consumption in the process of DNA calculation [27]. In [28], a symmetric key cryptosystem was designed by applying modern DNA biotechnology microarrays to cryptography. Both encryption and decryption keys are formed by DNA probes. The security of this algorithm depends on biological difficulties, so it is not affected by the changes of quantum computer attacks. In [29], a fast three-level DNA cryptography technique is displayed, which converts ciphertext information into DNA sequences by key shifting, complementary codes and twice DNA encoding with high encryption efficiency. In [30], the authors presented a data hiding method based on DNA coding. The addition operation is performed on the DNA sequence of the plaintext and the key, and the data is hidden by the cyclic movement of the entire sequence. The algorithm is better able to withstand violent attacks. In [31], based on attack prevention of DES and DNA computation encryption algorithm is put forward, using 128 nucleotides key replace 64 keys in DES algorithm, improves the ability of resisting violent attacks. In [32], a Telugu encryption method based on genetic DNA algorithm is proposed, which follows the genetic process to encrypt English text into Telugu characters, and has a good avalanche effect. In [33], the authors showed an asymmetric DNA encryption and decryption technique for the Arabic plaintext. The authors utilized a mixture of RSA, dynamic encoding and DNA computing techniques to encrypt messages with good randomness. Although DNA computing can reduce the time complexity of encryption systems, these algorithms have problems such as fixed DNA coding schemes and operating rules, high dependence on biology, and strict requirements on plaintext or ciphertext language types.

The characteristics of chaotic system, such as extreme sensitivity of initial value, and unpredictability of chaotic sequence [34], are consistent with many requirements of cryptography, so it is widely used in various encryption systems. In [35], the author came up with an improved One-Time-Pad (OTP) cipher algorithm, which uses random sequence generated by chaotic systems as the key to modularly encrypt each bit or character of the text. It reduces the difficulty of key generation of OTP and improves the randomness of key. In [36], the author introduced a method of text encryption for chaos theory and DNA computing. By means of hyperchaotic mapping, the plaintext is encrypted in two stages, namely bit-level permutation process and hyperchaotic sequence DNA coding replacement, which improves the robustness and has a large key space. In [37], an encryption method based on logistic map and three-dimensional matrix is displayed. The algorithm uses the shuffle of the three-dimensional matrix to change the position of the plaintext characters, and extends the small changes in a symbol to the entire ciphertext space through the diffusion mechanism, which improves the complexity of the ciphertext and the ability to resist violent attacks. In [38], the authors mentioned a new block cipher algorithm based on chaos. The new chaotic method based on multiplicative inverse function is used to control the diffusion of block cipher by chaotic system, which enhances the performance of logistic map and has strong key sensitivity. In [39], authors revealed a text encryption method using image encryption algorithm. This method converts text to image information, and uses the existing image encryption algorithm to encrypt, providing a new train of thought. Although the characteristics of chaotic system are reflected in the above algorithms, these algorithms are greatly influenced by the length of plaintext information as well as the high requirement of key randomness.

These encryption algorithms are rarely used in the medical field, and even less studied in stem cell bank. In order to address the issue of data leakage during the storage and transmission of private information in stem cell bank, this article proposed the double encryption protection (DEP) algorithm that based on improved AES and chaotic encryption technology. This algorithm ensures the security of privacy information storage and transmission, effectively safeguarding the confidentiality of the private data.

The main contributions of this study are as follows:

1. The key of each subsystem in the double encryption protection (DEP) algorithm is obtained by the proposed hash value key decomposition algorithm, which implements ‴one-time-one-secret", improves the key sensitivity and extends the key space. The presented two levels encryption protection algorithm enables dynamic encryption of flexible length messages and enhances the uncertainty and unpredictability of encrypted information.
2. The evaluation indexes of text encryption algorithms against differential attacks are displayed, namely the number of characters change rate (NCCR) and the uniform average changing intensity of text (UACIT), which can be applied to estimate the high sensitivity and security effectiveness of encryption algorithms for plain information.

The rest of this paper is organized as follows: Section 2 introduces the relevant theoretical basis. Section 3 describes a double encryption protection algorithm. Section 4 presents the simulation experiment and security analysis of the algorithm. Finally, Section 5 summarizes the paper.

## 2. Related works

### 2.1 Mapping between DNA and binary

The DNA molecule consists mainly of four types of nucleotides, which are A(adenine), G(guanine), C(cytosine), T(thymine), wherein A and T, G and C are complementary pairs [40]. Data in the computer is stored in binary form. In binary coding, 0 and 1 are complementary, 00 and 11 are complementary, and 01 and 10 are complementary [41]. The number of binary codes and bases are all four, and it can be assumed that the binary codes 00, 01, 10, 11 and bases A, T, C, and G satisfy a one-to-one mapping relationship. According to the coding rules, there are 4! = 24 coding schemes, but only 4×2 = 8 coding schemes that can satisfy the principle of base complementary pairing, and these 8 coding schemes are shown in Table 1. Assuming the binary representations for bases A, T, C, and G are 00, 10, 11, and 01, respectively. In this case, base A (00) is not complementary to base T (10) in binary, and base C (11) is not complementary to base G (01) in binary. Therefore, that coding scheme is not among the eight coding schemes proposed in Table 1.

**Table 1:** DNA coding scheme.

	1	2	3	4	5	6	7	8
A	00	00	01	01	10	10	11	11
T	11	11	10	10	01	01	00	00
C	01	10	00	11	00	11	01	10
G	10	01	11	00	11	00	10	01

As can be seen from Table 1, there is a one-to-one correspondence between binary sequence and base in each encoding scheme, that is, there are 8 encoding ways of binary sequence to base. Therefore, there are 8 decoding modes from base to binary sequence, which are shown in Table 2.

**Table 2:** DNA decoding scheme.

	1	2	3	4	5	6	7	8
00	A	A	C	G	C	G	T	T
11	T	T	G	C	G	C	A	A
01	C	G	A	A	T	T	C	G
10	G	C	T	T	A	A	G	C

### 2.2 DNA operation

In our proposed algorithm, a total of four DNA operation rule are used. The results of DNA-ADD (+), DNA-SUB(-), DNA-XOR(⊕), and DNA-XNOR(⊙) operations with different coding schemes are also different. The results of DNA-ADD, DNA-SUB, DNA-XOR and DNA-XNOR operations using coding scheme 1 are shown in Tables 3–6.

**Table 3:** DNA-ADD operation results.

+	A	T	C	G
A	A	T	C	G
T	T	G	A	C
C	C	A	G	T
G	G	C	T	A

**Table 4:** DNA-SUB operation results.

-	A	T	C	G
A	A	C	T	G
T	T	A	G	C
C	C	G	A	T
G	G	T	C	A

**Table 5:** DNA-XOR operation results.

⊕	A	T	C	G
A	A	T	C	G
T	T	A	G	C
C	C	G	A	T
G	G	C	T	A

**Table 6:** DNA-XNOR operation results.

⊙	A	T	C	G
A	T	A	G	C
T	A	T	C	G
C	G	C	T	A
G	C	G	A	T

### 2.3 Chaotic systems

#### 2.3.1 Logistic map

Logistic map is a typical one-dimensional chaotic map and one of the simplest and most studied nonlinear systems [42], which can be expressed as Eq (1):

$$x_{n+1}=\mu x_{n}(1-x_{n})$$ (1)

**Fig 1:**
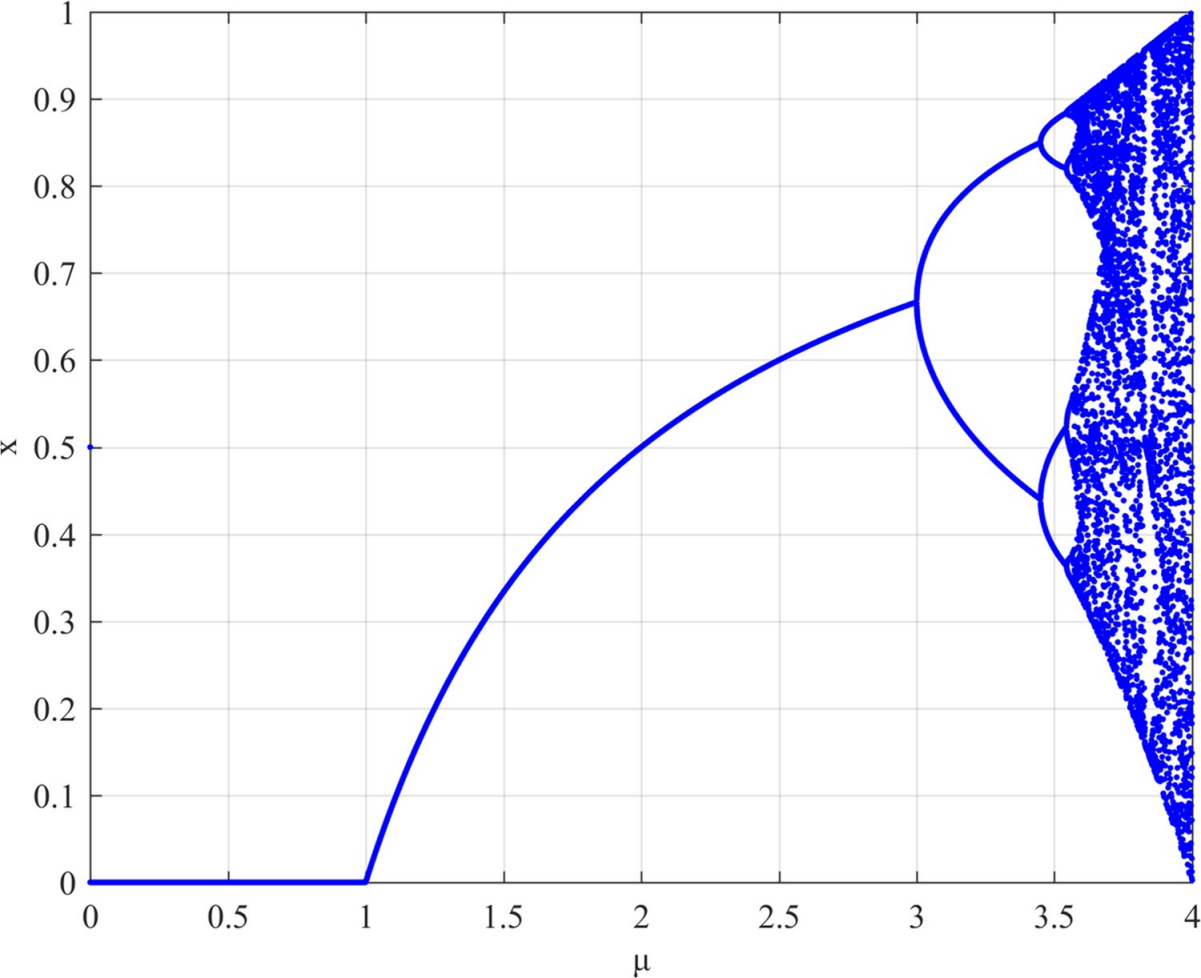
The bifurcation diagram of logistic map.

In Eq (1), μ is the parameter of the system. The bifurcation diagram illustrated in Fig 1 demonstrates the impact of varying the parameter μ in the logical mapping equation on the system’s behavior. The horizontal axis represents the values of parameter μ, while the vertical axis represents the values of the system state x. Within the range of (3.5699,4], the system’s state values exhibit chaotic behavior [43, 44], whereas in the remaining range, the system’s state values display periodic behavior. In our proposed algorithm, the initial value of logistic map is obtained by the hash value key decomposition, and the generated chaotic sequence will be used in the second level of encryption protection algorithm of privacy data of stem cell bank.

#### 2.3.2 Chen’s hyper-chaotic system

Chen’s hyper-chaotic system is widely used in encryption technology because of its unique high complexity and large key space [45]. Chen’s hyper-chaotic system can be expressed as Eq (2):

$$\{\begin{array}{c} \dot{x}=a(y-x)\\ \dot{y}=-xz+dx+cy-h\\ \dot{z}=xy-bz\\ \dot{h}=x+k \end{array}$$ (2)

**Fig 2:**
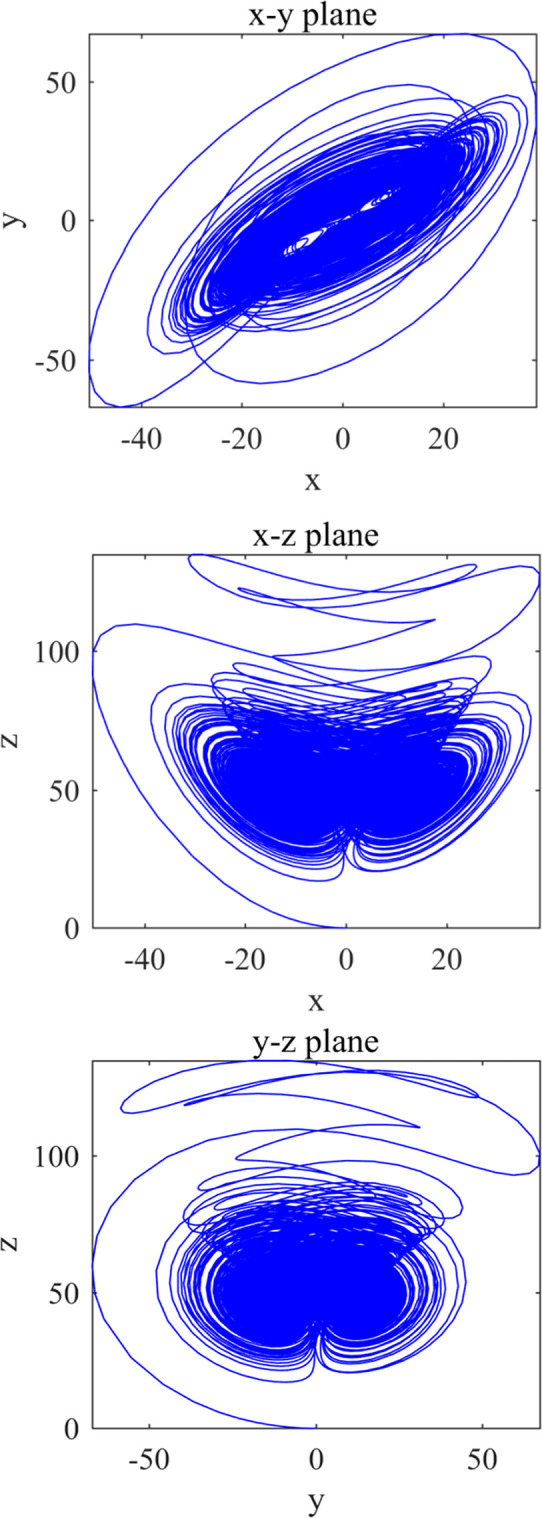
Attractors of Chen’s hyper-chaotic system (a) x-y plane (b) x-z plane (c) y-z plane.

In Eq (2), $\dot{x},\dot{y},\dot{z},\dot{h}$ are the differential states, and a, b, c, d, k are the system parameters. When a = 36, b = 3,c = 28, d = 16 and −0.7 ≤ k ≤ 0.7, Chen’s hyper-chaotic system is in hyper-chaotic state and can generate four chaotic sequences [46–48]. The chaotic attractor map, as depicted in Fig 2 provides valuable insights into the system’s dynamic behavior. Fig 2(A)–2(C) represents the attractors of the Chen hyper-chaotic system plotted in the x-y, x-z, and y-z planes, respectively. In these plots, the x-axis represents the first state variable of the system, while the y-axis represents the second state variable. By observing the shape and structure of the attractor, we find that Chen’s system exhibits good chaotic characteristics. Moreover, the properties of both the attractor and the resulting chaotic sequence are sensitive to changes in parameter values. Four initial values of Chen’s hyper-chaotic system are obtained by the hash value key decomposition, and the four chaotic sequences will be used in the second level of encryption protection algorithm of privacy data of stem cell bank.

## 3.The proposed algorithm

**Fig 3:**
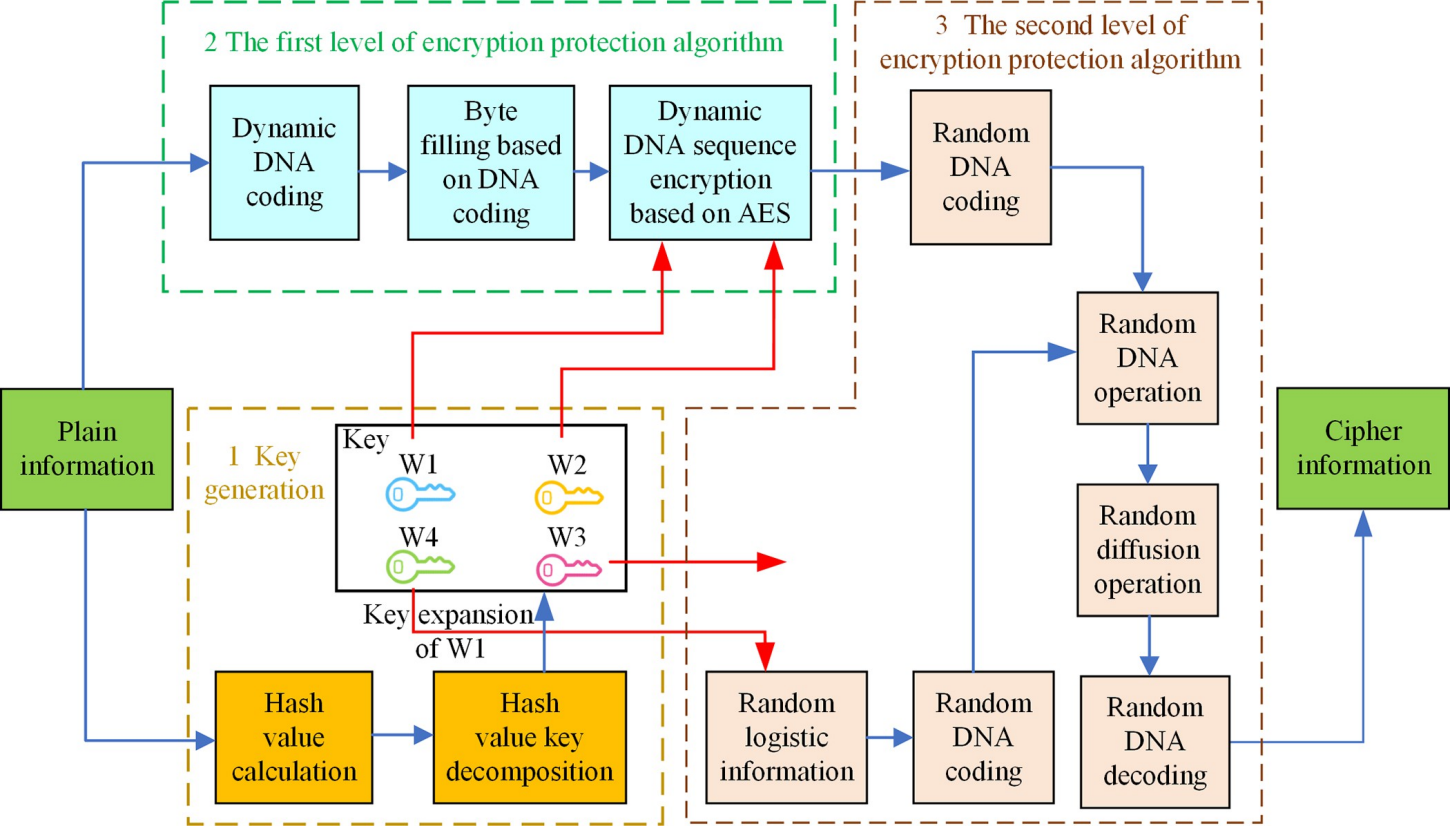
The structure of DEP algorithm.

The double encryption protection (DEP) algorithm is proposed based on improved AES and chaotic encryption technology for stem cell bank privacy data. Its structure is shown in Fig 3.

It can be seen from Fig 3 that the DEP algorithm is divided into three modules: key generation, the first level of encryption protection algorithm, and the second level of encryption protection algorithm. The stem cell bank privacy data encryption steps are as follows:

Step 1: The hash value of the plain information is calculated, and the key W1-W4 is obtained by hash value key decomposition, where the key W1 also requires key expansion.
Step 2: The plain information and the key W1 and W2 are used as input to participate in the first level of encryption protection operation.
Step 3: The results of step 2, the key W3 and the random logistic information generated according to the key W4 are used as input to participate in the second level of encryption protection operation to obtain cipher information.

The following subsections describe the above steps in detail.

### 3.1 Key generation

**Fig 4:**
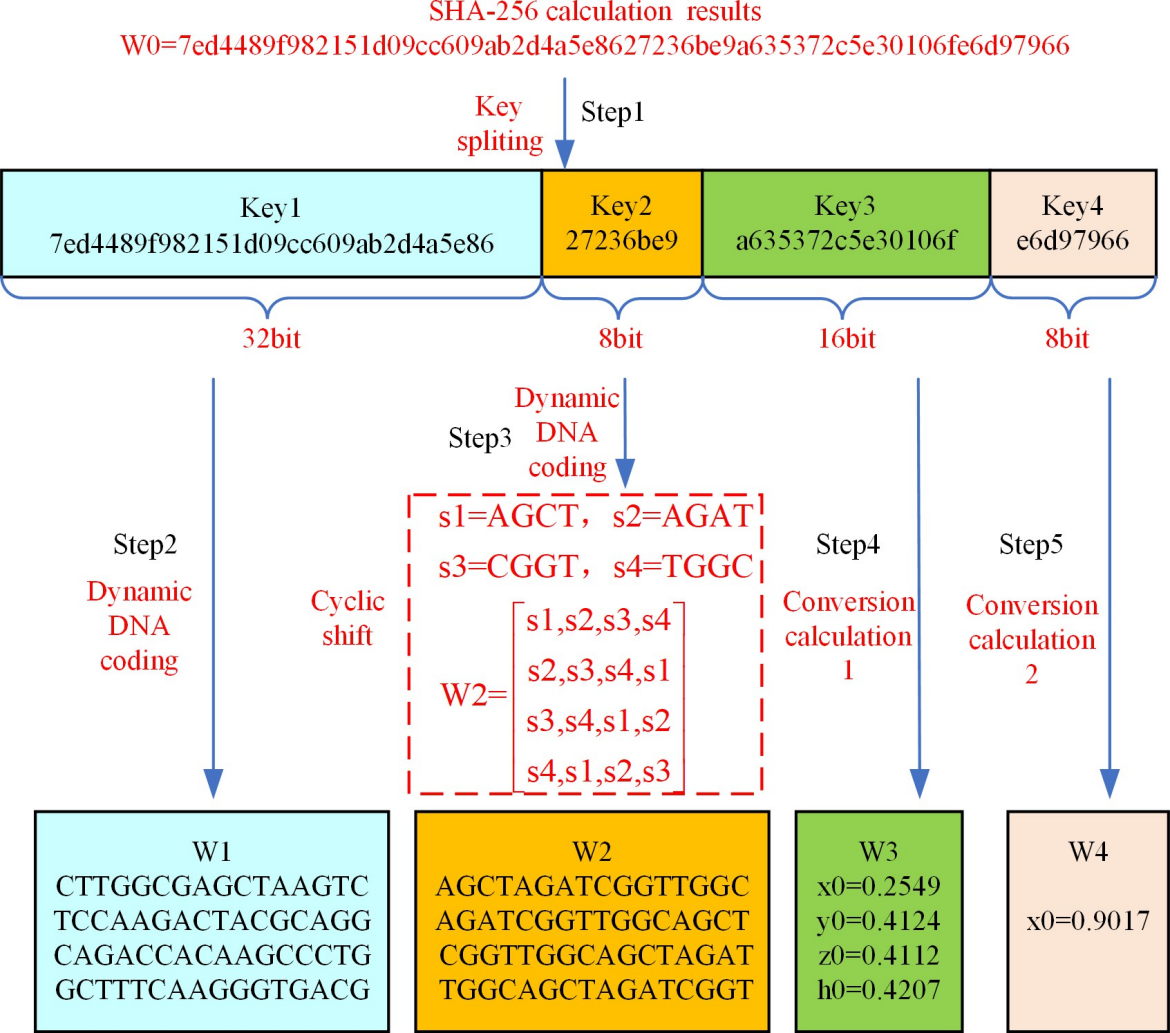
The process of hash value key decomposition.

The hash function SHA-256 [49] can convert information of different lengths into a 64-bit hexadecimal data. After inputting the plaintext information, the hash function generates a 64-bit data called W0 based on the content of the plaintext information. W0 is used as input to perform hash value key decomposition to obtain the key from W1 to W4. Fig 4 shows an example of the hash value key decomposition process.

As indicated in Fig 4, splitting W0 into varying lengths results in Key1, Key2, Key3, and Key4. After performing dynamic DNA coding on Key1, the key matrix W1 of the round key addition process of the first level of encryption protection algorithm is obtained. After dynamic DNA coding of every two bit of hexadecimal data in Key2, four DNA sequences s1-s4 are acquired. The left fixed matrix W2 of the mix columns process of the first level of encryption protection algorithm is gained by the cyclic shift of these four DNA sequences. After Key3 through the conversion calculation 1 from hexadecimal to decimal data, Chen’s hyper-chaotic system initial value W3 is acquired, in which W3 includes four data, x0, y0, z0 and h0. The initial value W4 of the logistic map is attained by using Key4 to proceed conversion calculation 2. The hash value key decomposition algorithm is shown in Algorithm 1:

**Algorithm 1** Hash value key decomposition

**Input:** W0

Step1: Key splitting

Key1 = *Split*(W0(1:32)); // *Split* is a partition function.

Key2 = *Split*(W0(33:40));

Key3 = *Split*(W0(41:56));

Key4 = *Split*(W0(57:64));

Step2: Dynamic DNA coding

W1 = *Code*(Key1,A); // *Code* is an coding function. A is the coding scheme

used, ranging from 1 to 8.

Step3: Dynamic DNA coding and cyclic shift

s1 = *Code*(*Split*(Key2(1:2)),A);

s2 = *Code*(*Split*(Key2(3:4)),A);

s3 = *Code*(*Split*(Key2(5:6)),A);

s4 = *Code*(*Split*(Key2(7:8)),A);

W2(1,:) = [s1,s2,s3,s4]; // The first row of W2 is arranged in the order of s1-s4.

W2(2,:) = [s2,s3,s4,s1];

W2(3,:) = [s3,s4,s1,s2];

W2(4,:) = [s4,s1,s2,s3];

Step4: Conversion calculation1

xt = *hex2dec*(*Split*(Key3(1:4))); //*hex2dec* is a conversion function from

hexadecimal to decimal.

x0 = *mod*(xt,10000)×10^-4^; //*mod* is the remainder function.

yt = *hex2dec*(*Split*(Key3(5:8)));

y0 = *mod*(yt,10000)×10^-4^;

zt = *hex2dec*(*Split*(Key3(9:12)));

z0 = *mod*(zt,10000)×10^-4^;

ht = *hex2dec*(*Split*(Key3(13:16)));

h0 = *mod*(ht,10000)×10^-4^; // W3 contains x0, y0, z0, h0.

Step5: Conversion calculation2

t1 = *hex2dec*(*Split*(Key4(1:4)));

t2 = *hex2dec*(*Split*(Key4(5:8)));

W4 = *mod*(*sum*(t1+t2),10000)×10^-5^; // *sum* is an accumulation function.

**Output:** W1, W2, W3, W4

According to the hash value key decomposition algorithm, the hash value W0 of the plain information will be divided into 4 parts. Key1 is the 1 to 32 bits of W0, Key2 is the 33 to 40 bits of W0, Key3 is the 44 to 56 bits of W0, and Key4 is the 57 to 64 bits of W0. Key1 selects a coding method from eight coding schemes and proceeds to dynamic DNA coding to obtain the key W1. Key2 also selects one of the eight coding schemes for dynamic DNA coding and performs circular left shift to get the key W2. Every four bits of data in Key3 is treated as a group, which carries out the hexadecimal to decimal conversion and the remainder operation. The resulting x0, y0, z0 and h0 are the key W3. Every four bits of data in Key4 is converted from hexadecimal to decimal as a group. The key obtained by accumulating and taking the remainder of the decimal data is W4.

Based on the key generation steps described in Fig 3, it is evident that the 4x16 matrix W1 shown in Fig 4 requires key expansion. This expansion is necessary to meet the requirements of the round key addition process in the DEP algorithm. The key expansion algorithm for W1 is shown in Algorithm 2.

**Algorithm 2** Key expansion

**Input:** W1(size:4×16)

W1 = [W1[1] W1[2] W1[3] W1[4]]

***for*** *i* = 5:1:44

***if*** (*i*-1) *mod* 4 = = 0

W1[*i*] = W1[*i*-4]⊕T(W1[*i*-1]);

***else***

W1[*i*] = W1[*i*-4]⊕ W1[*i*-1];

***end***

***end***

**Output:** W1(size:4×176)

**Fig 5:**
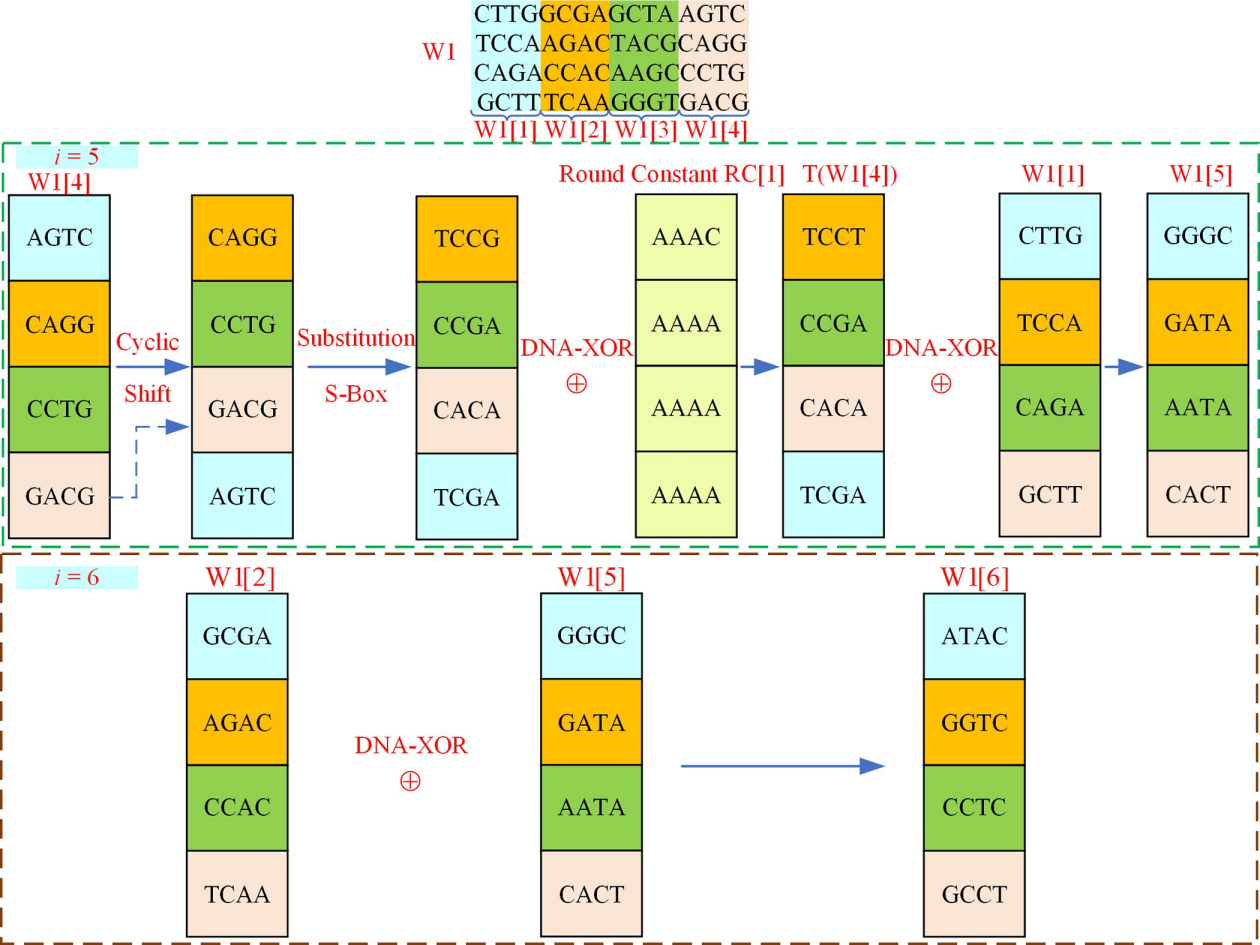
The process of key expansion.

According to Algorithm 2, W1 is a matrix with 4 rows and 16 columns at the beginning, and each 4 columns of DNA sequence in W1 can be divided as a group to get W1[1], W1[2], W1[3] and W1[4]. The new data generated in each expansion operation is denoted as W1[*i*]. The expansion operation starts from *i* = 5 and adds 1 each time until the end of *i* = 44. If (*i*-1) is a multiple of 4, we need to participate in the operation of the T-function before performing the DNA-XOR operation, otherwise we can perform the DNA-XOR operation directly. After the key expansion, W1 has a total of 44 groups of data, each group of data includes four columns of DNA sequences. Consequently, the output W1 has a total of 4 rows, 44×4 = 176 columns of data. An illustration of the key extension process is displayed in Fig 5.

It can be observed form Fig 5 that after splitting the original key W1, the data size of each block is 4×4. According to the key expansion algorithm shown in Algorithm 2, when calculating the key W1[5], *i* = 5, and *i*-1 = 4 is a multiple of four. Therefore, W1[5] needs to perform the T-function operation first, and then perform the XOR operation with W1[1]. The T-function operation includes three processes: cyclic shift, substitution bytes, and Round Constant DNA-XOR. The cyclic shift process moves the first row of data to the last row, with the remaining rows of data moving up one row. The data after the cyclic shift process needs to be replaced by the S-Box. The specific process of substitution S-box will be given in Section 3.2.3. Taking the data ’CAGG’ as an example, it becomes ’TCCG’ after being replaced by the S-box. The data replaced by the S-box requires an DNA-XOR operation with the Round Constant. At this time, the Round Constant is RC[1], and the result obtained by the completion of the T-function operation is T(W1[4]). The extended key W1[5] can be obtained by DNA-XOR operation of W1[1] and T(W1[4]). When calculating the key W1[6], *i* = 6, and *i*-1 = 5 is not a multiple of four. Therefore, the extended key W1[6] can be obtained by DNA-XOR operations on W1[2] and W1[5]. The Round Constant (RC) used in T-function operations is shown in Table 7.

**Table 7:** Round constant.

*j*	RC[*j*]	*j*	RC[*j*]	*j*	RC[*j*]	*j*	RC[*j*]	*j*	RC[*j*]
1	AAAC AAAA AAAA AAAA	2	AAAG AAAA AAAA AAAA	3	AACA AAAA AAAA AAAA	4	AAGA AAAA AAAA AAAA	5	ACAA AAAA AAAA AAAA
6	AGAA AAAA AAAA AAAA	7	CAAA AAAA AAAA AAAA	8	GAAA AAAA AAAA AAAA	9	ACGT AAAA AAAA AAAA	10	ATCG AAAA AAAA AAAA

As is shown in Table 7, we know that the data of RC[*j*] are different when the value of variable *j* is different. Furthermore, the relationship between variable *j* and variable *i* in key expansion satisfies the below Eq (3):

j=(i−1)/4 (3)

According to the value of variable *i* in T-function operation, the corresponding RC[*j*] is selected to complete the Round Constant DNA-XOR operation.

### 3.2 The first level of encryption protection algorithm

The first level of encryption protection algorithm includes three steps: dynamic DNA coding of plain information, byte filling based on DNA coding, and dynamic DNA sequence encryption based on AES. Among them, the dynamic DNA sequence encryption algorithm based on AES adopts the framework of AES encryption algorithm, contains four steps: substitution bytes, shift rows, mix columns, and add round key.

#### 3.2.1 Dynamic DNA coding

Plain information (input) is the private data of stem cell bank, which is one of the inputs of the first level of encryption protection algorithm. The plain information is converted to its corresponding ASCII value. Furthermore, one of the eight coding rules is randomly selected to apply dynamic DNA coding to ASCII values, and the data obtained after dynamic DNA coding is input1.

#### 3.2.2. Byte filling based on DNA coding

The length of data input1 encoded by dynamic DNA may not be an integer multiple of 64, and cannot participate in subsequent operations. As a consequence, before participating in dynamic DNA sequence encryption based on AES, it is necessary to do byte filling at the end to ensure that the data is 64 bits. The steps of byte filling based on DNA coding are as follows:

Step 1: The input1 encoded by dynamic DNA is grouped according to the 64-bit DNA sequence, and each block corresponds to a row of data.
Step 2: If the length of the last row of data is less than 64 bits, fill in the 4-bit DNA coding with the length divided by 4 after input1, meanwhile the remain data is filled with ’A’ until the length is 64 bits. If the length of the last row of data is 64 bits, fill in ’ACAA’ and sixty characters ’A’ after input1, totaling 64 bits of data.

**Fig 6:**
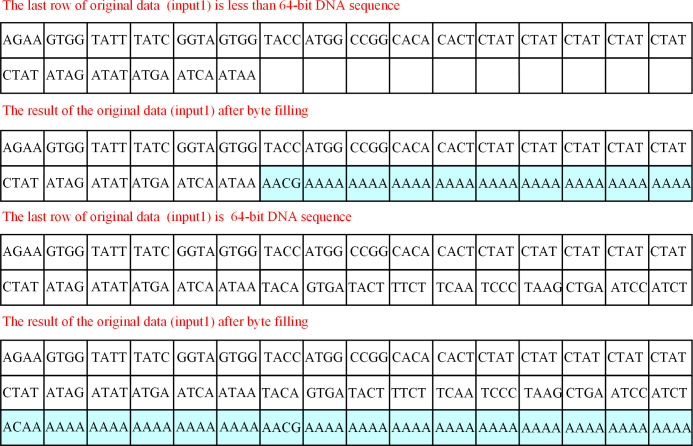
The process of byte filling based on DNA coding.

After byte filling based on DNA coding, obtain the data input1.The byte filling process illustrated is shown in Fig 6.

As can be seen from Fig 6, the original data (input1) is partitioned into two rows of data. According to DNA coding rule 1, if there are only 24 bits in the last row, DNA code ’AACG’ (24/4 = 6) needs to be filled in 25 to 28 bits, and character ’A’ needs to be filled in 29 to 64 bits. If the last row contains exactly 64 bits of data, fill in the first four bits of the new row with ’ACAA’ and the other 60 bits with character ’A’.

#### 3.2.3. Dynamic DNA sequence encryption based on AES

1. Substitution bytes

**Fig 7:**
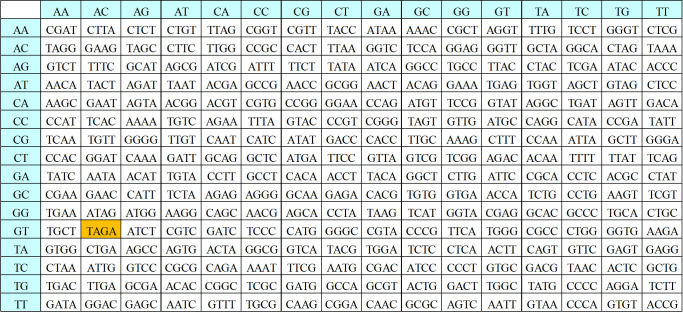
Substitution S-Box of DEP algorithm.

**Fig 8:**
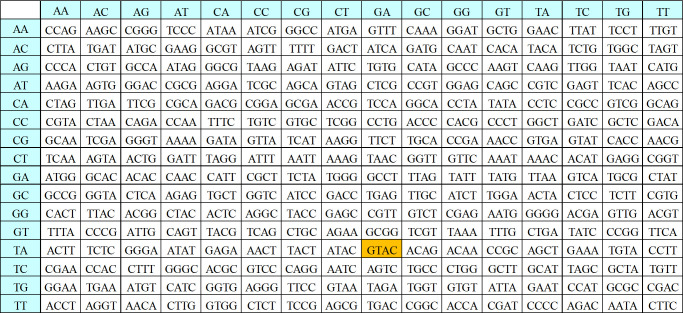
Substitution IS-Box of DEP algorithm.

The DEP algorithm redesigns the S-Box and Inverse S-Box (IS-Box) of the AES algorithm to accommodate substitution bytes in the proposed algorithm. The S-Box of the proposed algorithm is shown in Fig 7, and the IS-Box is shown in Fig 8.

From the Figs 7 and 8, it can be seen that the S-box and IS-box in the DEP correspond to the input of 4-bit DNA sequences and the output of 4-bit DNA sequences. The first two digits of the DNA sequence correspond to the number of rows, and the last two digits correspond to the number of columns. Taking ’GTAC’ as an example, the result after S-box operation is ’TAGA’, and the result after IS-box operation is ’GTAC’, which is successfully restored to the original data. Thereby proving that the IS-box is the inverse operation of S-box. The result of substitution the byte filled data (input1) with the S-box is input2.

2. Shift rows

At the beginning of the DEP shift rows, the original data (input2) after substitution bytes must be partitioned into blocks. The 16 blocks of data (input2) gained after block processing, each of block contains a 4-bit DNA sequence. The traditional AES algorithm has a fixed row shift scheme, which includes four schemes: no shift, cyclic left shift by one-bit, cyclic left shift by two-bis, and cyclic left shift by three-bit. In an effort to increase the randomness of the first level of encryption protection algorithm, the first row shift scheme of DEP algorithm selects one of the four schemes, the second row shift scheme selects one of the remaining three schemes, and so on. There are 4! = 24 options. The shift rows of DEP algorithm are shown in Algorithm 3, and the value of W0 is given in Fig 4 in Section 3.1.

**Algorithm 3** Shift rows

**Input:** input2, W0

Block input2

input2(1,:) = *Split*(input2(1:16));

input2(2,:) = *Split*(input2(17:32));

input2(3,:) = *Split*(input2(33:48));

input2(4,:) = *Split*(input2(49:64));

Shift rows scheme selection

A = *Strcat*(W0(5), W0(1)) = ’47’ // *Strcat* is a string contiguous function. The

result of extracting the fifth and first elements in W0 is A1.

A1 = *mod*(*hex2dec*(A), 24)+1 = 24 // Convert hexadecimal data A to decimal data.

The range of A1 is 1 to 24.

Shift rows according to the scheme A1

input3(1,1:16) = input2(1,1:16)

input3(2,1:16) = *Strcat*(input2(2,5:16), input2(2,1:4));

input3(3,1:16) = *Strcat*(input2(3,9:16), input2(3,1:8));

input3(4,1:16) = *Strcat*(input2(4,13:16), input2(4,1:12));

**Output:** input3

**Fig 9:**
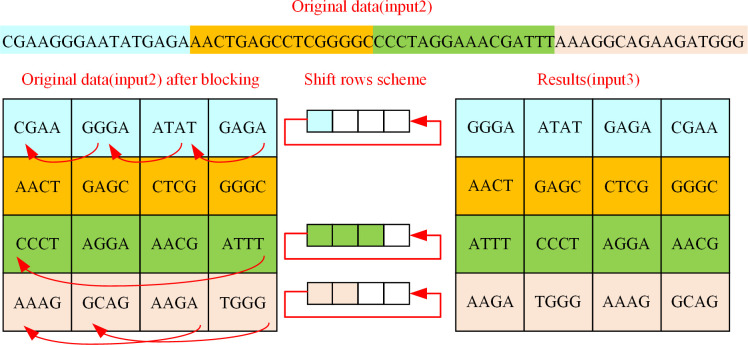
A kind of shift rows operation of DEP algorithm.

In the light of Algorithm 3, the input of the shift rows algorithm is the original data (input2) and W0. The new hexadecimal data A extracted and combined from the 5th and 1st characters in W0 is ’47’, and the A1 got by converting A into decimal and performing a remainder operation with twenty-four is 24. Thus, the 24th shift rows scheme is selected, that is, the first row is not shifted, the second row is shifted 1 bit to the left, the third row is shifted 2 bits to the left, and the third row is shifted 3 bits to the left. An example of the DEP shift rows process is shown in Fig 9.

As is shown in Fig 9, every 16-bit DNA sequence in the original data (input2) needs to be partitioned as a group. Among them, 1 to 16 bits of original data are used as the first row after the block, 17 to 32 bits of original data are used as the second row after the block, and so on to obtain the original data after the block (input2). At this time, the shift scheme is cyclic shift of the first row to the left by one bit, the second row without shift, the third row cyclic shift to the left by three bits, and the fourth row cyclic shift to the left by two bits. The result of the shift according to the shift rows scheme is input3.

3. Mix columns

**Fig 10:**
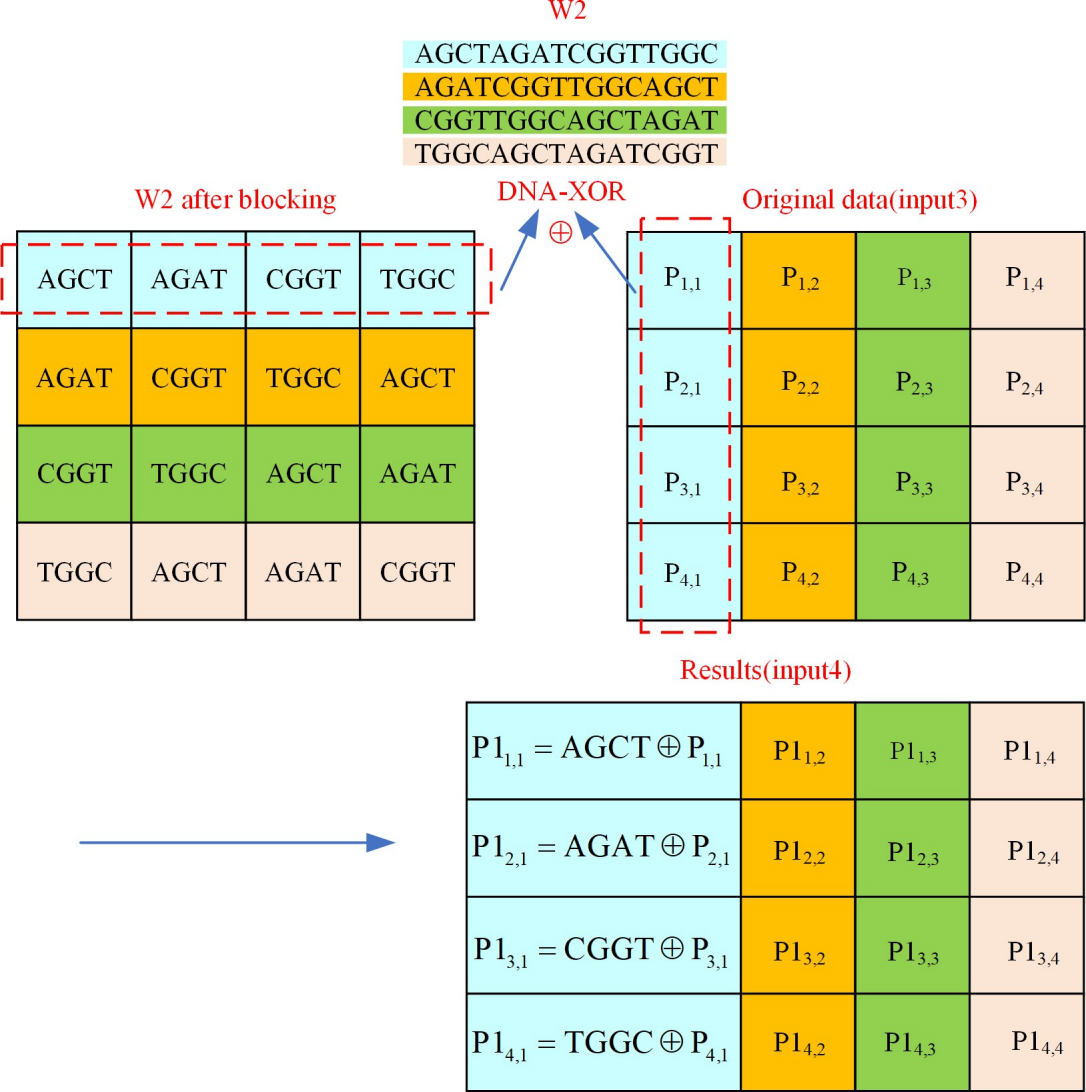
Mix columns operation of DEP algorithm.

The mix columns operation of the AES algorithm requires two types of operations: multiplication and XOR. In the cause of simplify the mix column calculation steps and improve encryption efficiency, the mix column in the DEP algorithm only requires the DNA-XOR operation. The fixed matrix on the left side during mixing columns is W2 obtained after the hash value key decomposition, and this W2 with the original data (input3) after shifting rows are used as the two inputs in the mix columns process. An explanation of the DEP algorithm for mixing columns operation is provided in Fig 10.

It is clear from Fig 10 that every 4 column of DNA data in the key W2 (size: 4×16) are chunked as a group, and a total of 16 blocks of data are acquired. The mix columns process is to conduct the DNA-XOR operation between the blocked key W2 and the original data (input3), where the value of the key W2 has been given in Fig 4 in Section 3.1. For instance, the data P1_2,1_ in the mixing columns result (input4) is the result of the DNA-XOR of the base sequence ’AGAT’ with position coordinates (1,2) in W2 and the data P_2,1_ with position coordinates (2,1) in input3, and similarly the mix columns result (input4) of all data can be acquired.

4. Add round key

**Fig 11:**
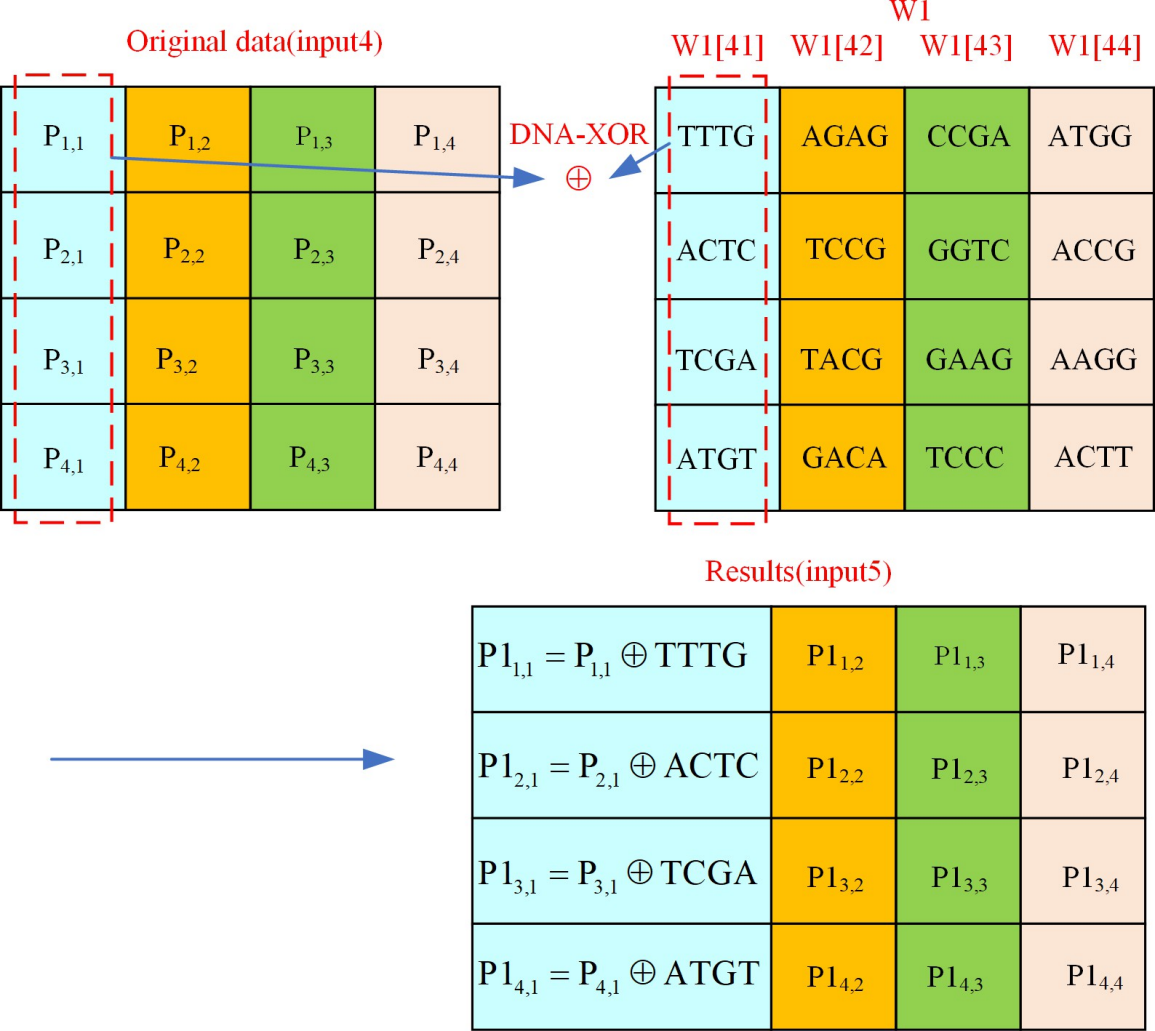
Add round key operation of DEP algorithm.

The input of adding round key in DEP algorithm are the original data (input4) received after mixing columns and the key W1, and each calculation uses 4 columns of W1 data. The add round key process described as an example is shown in Fig 11.

As shown in Fig 11, the data in columns 41 to 44 of key W1 are involved in this add round key operation. Consider the data P1_2,1_ in the add round key result (input5) as an example, it is the result of DNA-XOR on the data P_2,1_ with position coordinates (2,1) in input4 and the DNA sequence ’ACTC’ with position coordinates (2,1). Accordingly, the result (input5) of the add round key of all the data is available. At this point, the first level of encryption protection algorithm ends and the resulting first level of encryption protection cipher information is input5.

### 3.3 The second level of encryption protection algorithm

In order to further improve the security of the privacy data of the stem cell bank, the cipher information (input5) obtained after the first level of encryption protection needs to be encrypted in the second level. The extreme sensitivity and unpredictability of initial values of chaotic systems are well suited for encryption of privacy data. However, the structure of low-dimensional chaotic graphs is relatively simple and less secure [50, 51]. In contrast, the Chen’s hyper-chaotic system exhibits strong chaotic behaviour and has complex dynamic properties, which makes it a favourable choice among encryption algorithms [47, 49]. The complexity and unpredictability of Chen’s hyper-chaotic system can enhance the security of the algorithm. Therefore, in the second level of the encryption protection algorithm, we use the Chen’s hyper-chaotic system as the basis for encryption. The random sequence generated by the Chen’s hyper-chaotic system is used to determine the exact scheme of the encoding, operation, diffusion and decoding processes of the second level of the encryption protection algorithm. This increases the security of the private data of the stem cell bank.

The key W4 generated after the hash value key decomposition is applied as the original value of the logistic mapping, and the random logistic information (input6) is generated through the logistic mapping. The algorithm for generating the random logistic information is demonstrated in Algorithm 4:

**Algorithm 4** Random logistic information generation

**Input:** W4, input5

MUL = M×N; // M and N are the number of rows and columns of the input5

matrix of the first level of encryption result.

P0 = 1500;

***for*** *i* = 1:1:(P0+MUL)

P(*i*) = *Logstic*(W4); // The logistic sequence P is created using Eq (1).

***end***

P = *Split*(P(1501: end)); // Remove the first 1500 iterations of logistic chaotic to

eliminate the undesirable effects.

input6 = *mod*(*Ceil*(P×10^3^),256); // *Ceil* is an upward integer function. The range

of input6 is 0-255.

**Output:** Random logistic information(input6)

**Fig 12:**
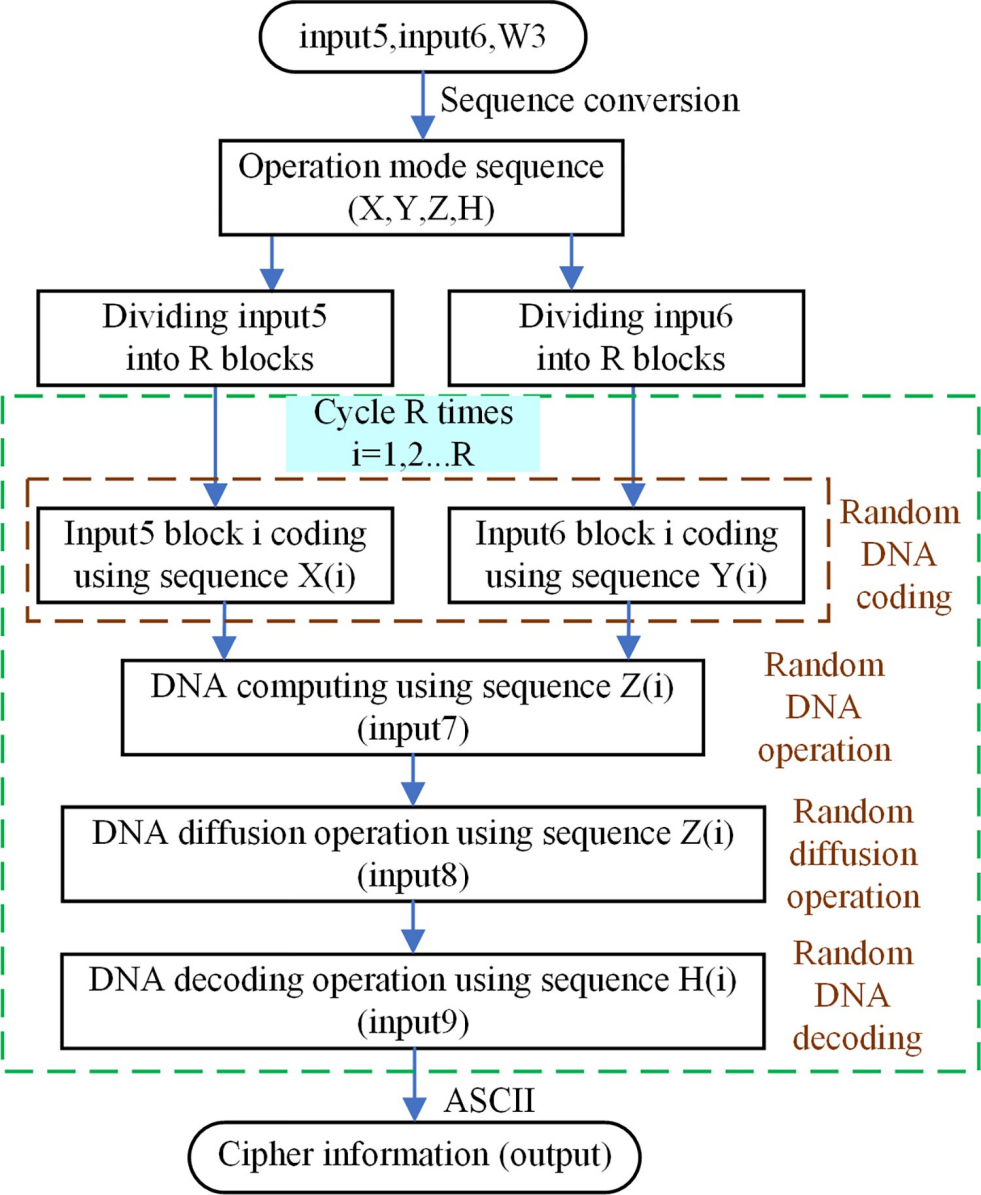
The second level of encryption protection algorithm of DEP.

It is known from the random logistic information generation algorithm that the logistic sequence P generated according to Eq (1) needs to discard the first 1500 terms to achieve better randomness. The ASCII range of the generated logistic sequence information (input6) is 0–255, which is the same size and ASCII range as the cipher information (input5) generated by the first level of encryption protection algorithm. The cipher information (input5), random logistic information (input6) and the key W4 generated after hash key decomposition are applied as input to the second level of encryption protection algorithm. The process of the second level of encryption protection algorithm is presented in Fig 12.

As observed from Fig 12, the second level of encryption protection algorithm needs to first create a sequence of operation modes based on the sequence conversion algorithm to confirm which operation mode is used in the subsequent encryption. The sequence conversion algorithm is shown in Algorithm 5.

**Algorithm 5** Sequence conversion

**Input:** W3, input5

P1 = 1500;

R = M×N/16; // M and N are the number of rows and columns of the input5

matrix of the first level of encryption result.

***for*** *i* = 1:1:(P1+R)

A(*i*, :) = *Chen_chaotic*(W3); // The Chen’s hyper-chaotic sequence A is created

using Eq (2).

***end***

X1 = A(:,1); // X1 is all the elements of the first column of the matrix A.

Y1 = A(:,2);

Z1 = A(:,3);

H1 = A(:,4);

X1 = *Split*(X1(1501:end)); // Remove the first 1500 iterations of hyper-chaotic to

eliminate the undesirable effects.

Y1 = *Split*(Y1(1501:end));

Z1 = *Split*(Z1(1501:end));

H1 = *Split*(H1(1501:end));

X = *mod*(*Floor*(X1×10^4^),8); // *Floor* is an downward integer function. The range

of X is 0 to 7.

Y = *mod*(*Floor*(Y1×10^4^),8);

Z = *mod*(*Floor*(Z1×10^4^),4);

H = *mod*(*Floor*(H1×10^4^),8);

**Output:** Operation mode sequence (X, Y, Z, H)

As we can see from Algorithm5, the range of X and Y after sequence conversion is 0–7 corresponding to 8 DNA coding methods, the range of Z is 0–3 corresponding to 4 DNA operation methods, and the range of H is 0–7 corresponding to 8 DNA decoding methods.

After sequence conversion, the cipher information (input5) generated by the first level of encryption protection algorithm and the random logistic information (input6) need to be split into blocks, both of which can be divided into *R* blocks because they are of the same size. The number of block *R* can be indicated as:

$$R=\frac{M\times N}{16}$$ (4)

In Eq (4), M and N refer to the number of rows and columns of input5, respectively. After splitting, input5 and input6 are scheduled for random DNA coding, random DNA operation, random diffusion and random DNA decoding, each time only for the *i-th* block of data, and a total of R cycles are necessary.

The operation mode sequence X(*i*) decide the coding method of the *i-th* block in input5, the operation mode sequence Y(*i*) determine the coding method of the *i-th* block in input6. Meanwhile, the operation mode sequence Z(*i*) dictate the operation method of the *i-th* block in input5 and the *i-th* block in input6, and the outcome is input7 after random operation. Moreover, the diffusion operation of the *i-th* segment is to diffusion operate the input7 of the *i-th* segment with the input7 of the (*i*-1)*-th* segment to get input8, where the operation mode of random diffusion is dictated by the operation mode sequence Z(*i*). The decoding mode of the *i-th* block in input8 depends on the operation mode sequence H(*i*), whose decoded value is input9. After DNA decoding, the data input9 has been converted to data in the range of [0,255], and the output of the second level of encryption protection algorithm (output) is gained by ASCII conversion of these data. At this point, the second level of encryption protection algorithm is finished, and the data (output) is the result of stem cell bank privacy data encrypted by the DEP algorithm.

## 4 Results and security analysis

**Fig 13:**
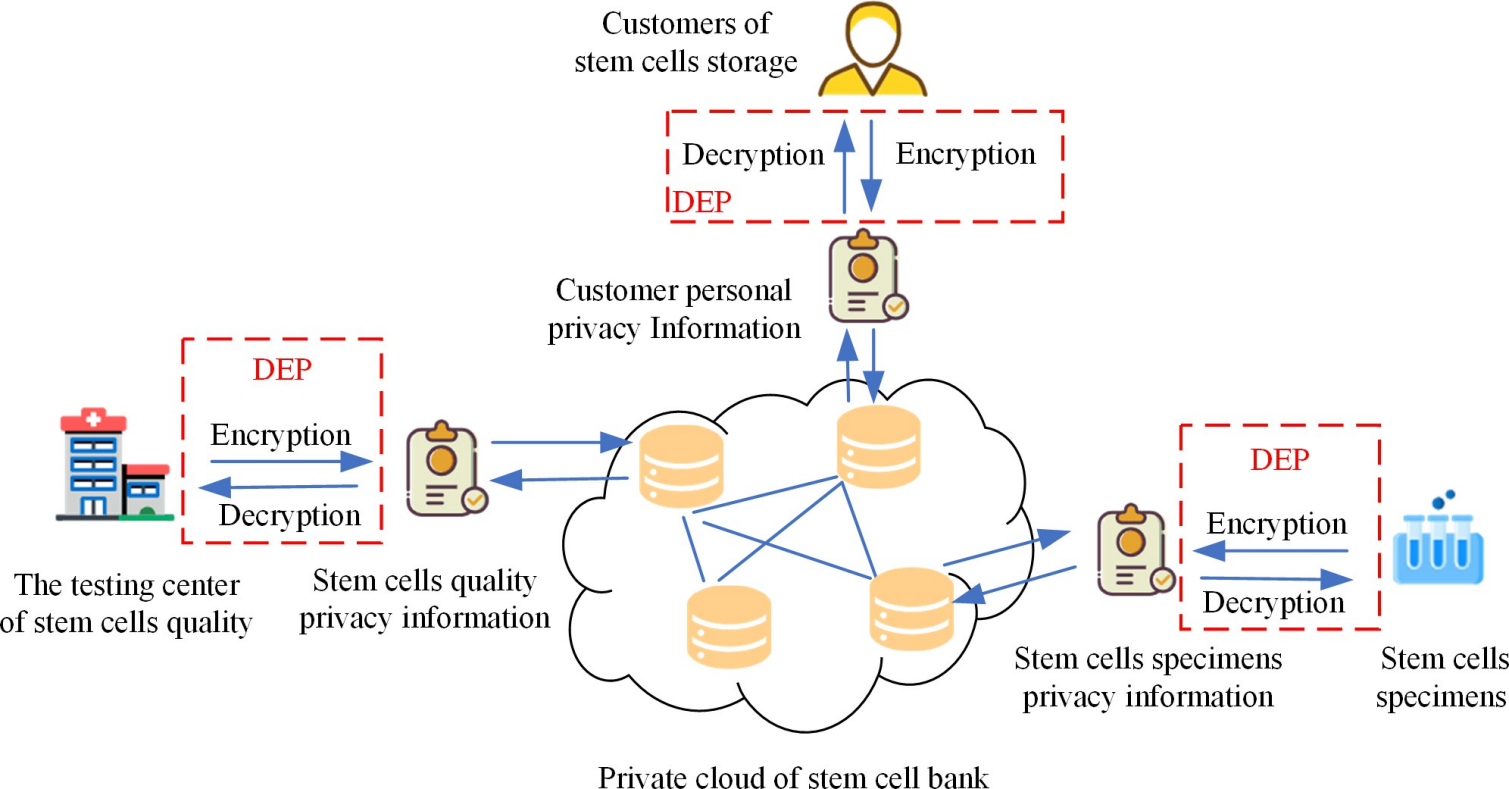
Private cloud access information system for stem cell bank private data.

In this section, we illustrate the security results of encryption stem cell bank privacy information using different algorithms. The privacy information is divided into three categories according to the source: the first category is the personal information of the customers who store the stem cells, the second category is the information of stem cell specimens, and the third category is the information of stem cell quality issued by the quality testing center. These three types of private information are encrypted and saved in the private cloud of the stem cell bank. The private cloud access information system for stem cell bank privacy data is displayed in Fig 13.

As can be seen from Fig 13, personal privacy information, stem cell sample privacy information as well as stem cell quality privacy information are generated by the DEP algorithm. The above information are decrypted by DEP and then the plaintext information of the corresponding privacy data can be obtained. In the following, we test the encryption of customer personal information, stem cell specimen information as well as stem cell quality information using five encryption methods: DES, AES, encryption based on DNA computation and hyperchaotic system proposed in the literature [36], AES encryption based on DNA proposed in the literature [52], and our proposed DEP encryption. The security of the algorithms is also evaluated in five aspects: histogram, information entropy, key space, key sensitivity and differential attack.

In this experiment, a laptop with Core i5-1135G7 2.4GH CPU, 16GB RAM, and Windows 10 operating system was used to simulate and evaluate the proposed algorithm using MATLAB R2017a. All the test information used in this section is displayed in Table 8. In Table 8, the three text information categorized according to the source of privacy information are Text1, Text2 and Text3.

**Table 8:** Test information.

Text	Information
Text1	母亲姓名:李XX 联系方式:152xxxx9567 身份证号码:41578******1547607 合同号:ZDGssssss23840(Chinese)
Text2	河南郑大干细胞库科技有限公司xxxx干细胞标本信息 采集地址:河南省****医院 标本数量:**** 新生儿出生日期:2022.09.** 标本接收时间:2022.09.** 15:04 标本接收人:李XX(Chinese)
Text3	1.冻存细胞管数:s 2.血液免疫学检测:(1)乙肝病毒表面抗原:阴性 (2)丙型肝炎病毒抗体:阴性 (3)艾滋病病毒抗体:阴性 (4)巨细胞病毒抗体:阴性 (5)梅毒螺旋体抗体:阴性 3.无菌检测:(1)需氧菌:阴性 (2)厌氧菌:阴性 4.支原体检测:阴性 5.内毒素检测:合格 6.细胞活性:98% 7.细胞形态学检测:正常 8.细胞表型:合格 9.储存温度:-196℃(Chinese)

Due to the fact that all the information in the stem cell bank is highly confidential and private, for security reasons, some symbols in Table 8 are used to replace the original content in the process of presentation to complete the de-privatization process.

### 4.1 Analysis of histograms

**Fig 14:**
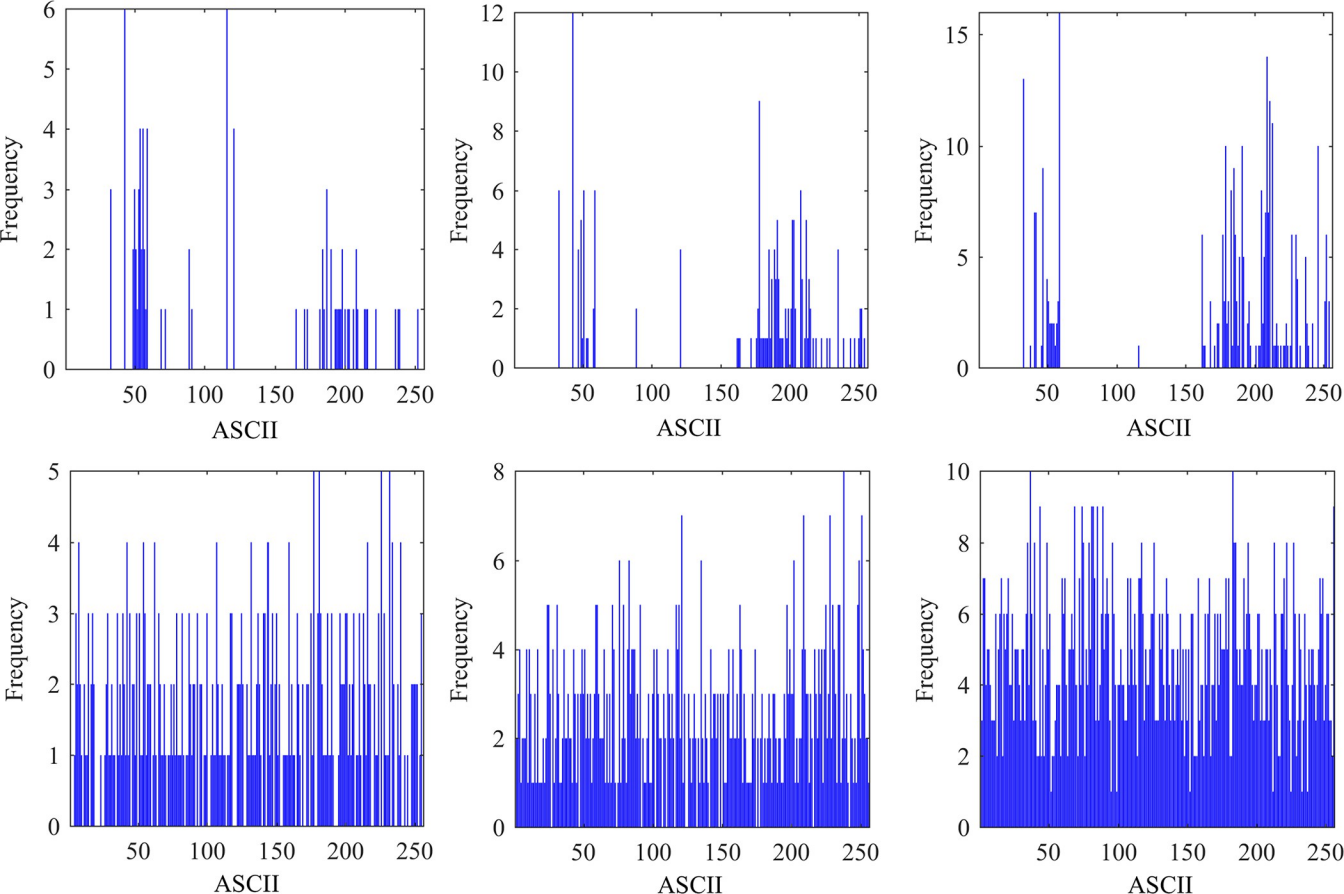
Histogram analysis (a)Text1 histogram (b) Text2 histogram (c)Text3 histogram (d)Text1 histogram encrypted by DEP algorithm (e)Text2 histogram encrypted by DEP algorithm (f)Text3 histogram encrypted by DEP algorithm.

A simple measure to evaluate the security of an encryption algorithm is to perform a histogram analysis. The more uniform the histogram distribution, the more difficult it is for an attack to infer the corresponding plaintext message based on the character distribution regularities [53, 54]. In the histogram evaluation of text information, the ASCII values corresponding to the characters are presented, and the histogram of plaintext and ciphertext messages is shown in Fig 14.

In Fig 14, the horizontal axis indicates the ASCII value of the character, and the vertical axis indicates the frequency of that character’s appearance. Moreover, (a)-(c) are the histograms of plaintext messages, and (d)-(f) are the histograms of ciphertext messages obtained by applying DEP, respectively. From Fig 14, it can be seen that characters with ASCII values below 50 and 100–150 range appear less frequently in plaintext messages, and the histogram distribution is more discrete and has some breakpoints. The ASCII values of ciphertexts generated by the DEP are more evenly distributed in the range of [0,255], and the longer the plaintext message is, the fewer breakpoints there are in the histogram of ciphertexts after encryption. By comparing the histograms before and after encryption, it is revealed that the original information can be effectively hidden after encryption by the DEP algorithm. For more intuitive evaluation of the uniformity of the histogram distribution, we calculate the variance of the histogram using the following variance Eq (5):

$$\mathrm{var}(x)=\frac{\sum_{i=1}^{N}{\left(x_{i}-\bar{x}\right)}^{2}}{N}$$ (5)

In Eq (5), *x* is the ASCII value of the character and *N* is the length of the character. The lower the variance, the more uniform the distribution of the encrypted characters, and the more unlikely it is for the attacker to break the information using statistical attacks. The variance of the cipher information obtained using the above formula is shown in Table 9.

**Table 9:** The variance of cipher information.

Text	DES	AES	Ref [36]	Ref [52]	Proposed
Text1	126.3864	122.3542	125.2093	121.1146	119.4245
Text2	123.1875	121.1813	121.3590	120.0938	116.6641
Text3	122.9638	120.1711	120.3333	120.0757	114.2763
Average	124.1792	121.2355	122.3005	120.4280	116.7883

As we can see from Table 9, the variance of the encrypted histogram is different for different lengths of text messages. With a fixed length of text message, the smaller variance of the encrypted histogram indicates the more uniform distribution of the encrypted message. When comparing the mean values of the histograms of each encryption algorithm, it can be found that the maximum variance value is 124.1792 for the DES algorithm encryption and the minimum variance value is 116.7883 for the DEP algorithm encryption, with a variance reduction of 5.95%. Consequently, the distribution of ASCII values of characters after encryption by DES algorithm is more discrete and the distribution of characters after encryption by our proposed algorithm is more uniform. As a result, it is proved that DEP algorithm is more resistant to statistical attacks and has the certain security.

### 4.2 Analysis of information entropy

Information entropy is considered as a measure of randomness [32, 55], with higher information entropy proving a higher degree of randomness of the encrypted message, which is calculated by the following Eq (6):

$$H(x)=-\sum_{i=1}^{N}T(x_{i})\log_{2}T(x_{i})$$ (6)

In Eq (6), *T*(*x*_*i*_) is the probability of occurrence of character *x*_*i*_, the probability of occurrence of a certain character ASCII value. The information entropy of the plain and cipher information obtained by the above formula is given in Table 10.

**Table 10:** Information entropy.

Text	Plain	DES	AES	Ref [36]	Ref [52]	Proposed
Text1	5.2630	6.1412	6.2854	6.0919	6.1109	7.4375
Text2	5.5199	6.7686	6.8750	6.8943	6.8436	7.6953
Text3	5.7085	7.3553	7.3615	7.3682	7.3173	7.8736
Average	5.4971	6.7550	6.8406	6.7848	6.7573	7.6688

It can be seen from Table 10 that the information entropy of the plain information is lower than that any of the encryption algorithms, indicating that the ciphertext message generated by these encryption algorithms has a certain degree of randomness. From the literature [56, 57], it is known that the desirable value of information entropy is 8. When the entropy value is closer to 8, it is regarded as having good randomness. By contrasting the mean values of information entropy, it can be seen that the text encryption method of DES has low uncertainty of the encrypted message. After applying our proposed DEP algorithm to encrypt three types of text information, the information entropy is improved and is closer to the ideal value of information entropy. The average value of information entropy is improved from the lowest 6.7550 to 7.6688, which is an improvement of 13.53%. Thereby, it is proved that our proposed algorithm guarantees the generation of cipher information with high randomness.

### 4.3 Analysis of key space

A secure and effective algorithm for stem cell bank privacy data should have a large key space in case the attacker can successfully restore the corresponding privacy information through brute force attack methods [58, 59]. Our proposed DEP algorithm performs a total of three dynamic encodings and one shift rows scheme selection in the first level of encryption. A total of two random encodings, one random operation, and one random decoding scheme selection are performed in the second level of encryption. The actual key parameters of the algorithm and the corresponding key space are shown below:

1、Key parameter of the first level encryption *S*_*f*_

$$S_{f}=S_{f1}\times S_{f2}=8^{3}\times 4!=3\times 2^{12}$$ (7)

2、Key parameter of the second level encryption *S*_*s*_

$$S_{s}=S_{s1}\times S_{s2}\times S_{s3}=8^{2}\times 4\times 8=2^{11}$$ (8)

3、Key parameters of system *S*_*k*_

$$S_{k}=16^{64}=2^{256}$$ (9)

In summary, the total key space of algorithm is:

$$S=S_{f}\times S_{s}\times S_{k}=3\times 2^{12}\times 2^{11}\times 2^{256}=3\times 2^{279}$$ (10)

The key space for various types of encryption algorithms is shown in Table 11.

**Table 11:** Key space.

DES	AES	Ref [36]	Ref [52]	Proposed
2^56^	2^128^	10^26^	2^131^	3×2^279^

From Table 11, we can see that DES requires a maximum of 2^56^ attempts to obtain the correct key. AES algorithm using 128-bit key encryption requires a maximum of 2^128^ attempts to get the information cracked. The text encryption algorithm based on DNA computation and hyperchaotic systems takes 10^26^ attempts to break. The AES algorithm based on DNA encoding takes 2^131^ attempts to decrypt. The key space of DEP algorithm is 3×2^279^, which has been improved to a great extent compared with several encryption algorithms mentioned above. To ensure the security of the encryption algorithm, the size of the key space should not be less than 2^128^ [60, 61]. Since the DEP algorithm has a large key space, the algorithm provides an effective defense against exhaustive attacks.

### 4.4 Analysis of key sensitivity

For the sake of avoiding attackers using similar keys to destroy the algorithm, the encryption system should have a certain key sensitivity to ensure that the plain data cannot be recovered correctly even after small changes. The key of DEP system is generated by hash algorithm, so it is very sensitive to the initial conditions, so any slight change will bring a big difference to the key data of each part of the system. Let’s take Text1 as an instance, the result of decryption using the correct key W1 is given in Table 12, and we change the last base ’G’ in the key to ’C’, and the decryption result obtained is given in Table 12.

**Table 12:** Analysis of key sensitivity.

Key	Decryption results
CTTGGCGAGCTAAGTC TCCAAGACTACGCAGG CAGACCACAAGCCCTG GCTTTCAAGGGTGACG	母亲姓名:李XX 联系方式:152xxxx9567 身份证号码:41578******1547607 合同号:ZDGssssss23840(Chinese)
CTTGGCGAGCTAAGTCTCCAAGACTACGCAGG CAGACCACAAGCCCTGGCTTTCAAGGGTGACC	颠蠭 脽嬄漆 = YF胧(貐^麿矬 鑢P*蚈 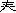 H稺 p埧ZI盱酮H Zx哥篤O 8苢BL 7 事翚 攙(Garbled information)

By analyzing Table 12, we can see that decrypting the key with slight modification will result in a large number of garbled information. The key W1 plays a key role in the key expansion process and in the round key addition process. It determines the subkeys used in each encryption round, and even a slight modification of W1 will result in a completely different subkey. By changing key W2, it leads the mixing columns operation using different matrices. The key W3 is responsible for generating the Chen’s hyper-chaotic sequence, which is utilized in blocking, coding, operation and decoding. Similarly, changing W4 will result in generating a different sequence of logic maps, causing changes in the data involved in DNA computing. In conclusion, the keys W1, W2, W3 and W4 have a key role in the different stages of encryption. Any modification of these keys will disrupt the corresponding procedures and sequences, making the decryption process unable to recover the original information correctly. In other words, our proposed DEP algorithm has extremely high key sensitivity.

### 4.5 Analysis of differential attack

Currently, the avalanche effect is used to measure the significant changes in text output results caused by the flipping of a bit of binary data in the text input data [25, 62]. However, as the length of the text message continues to increase, the complexity and computational effort of the avalanche effect calculation increases, making it unsuitable for reflecting the changes in ciphertext caused by plaintext modifications in our proposed DEP algorithm. The NPCR (Number of pixels change rate) and UACI (Uniform average change intensity) metrics are widely used to evaluate pixel differences before and after encryption in the field of image encryption [63, 64]. They can detect pixel-level changes, such as individual pixel modifications or flipping of a few pixels. This enables them to effectively assess the avalanche effect of encryption algorithms and quantify the degree of impact on images, without considering the size of the images when calculating NPCR and UACI. Therefore, we introduce the NCCR (Number of characters change rate) and UACIT (Unified average change intensity of text) metrics in our text encryption research to evaluate the impact of plaintext changes on ciphertext. The core concept is based on the widely-used evaluation metrics NPCR and UACI in the field of image encryption, which measure the differences in ciphertext information under different inputs. Compared to existing metrics, the proposed NCCR and UACIT simplify the calculation of ciphertext changes due to plaintext modifications, while also calculating the average change intensity of text data. NCCR and UACIT are calculated as follows:

$$E(i)=\{\begin{array}{c} 0\;if\;A(i)=B(i)\\ 1\;if\;A(i)\neq B(i) \end{array}$$ (11)

$$NCCR=\frac{1}{N}\sum_{i=1}^{N}E(i)\times 100(\%)$$ (12)

$$UACIT=\frac{1}{N}\sum_{i=1}^{N}\frac{\left|A(i)-B(i)\right|}{255}\times 100(\%)$$ (13)

In the above equation, *A*(*i*) represents the ASCII value of the cipher information generated from the unmodified plain, *B*(*i*) represents the ASCII value of the cipher information generated from the modified plain with a small number of characters, and *N* is the length of the ciphertext message. NCCR and UACIT indicators reflect the sensitivity of the encryption algorithm to changes in the plaintext message, whereby higher values of these two indicators indicate that the encryption system is more resistant to differential attacks. The NCCR and UACIT indicators obtained by using the above formula are shown in Table 13, where the plain information used in different algorithms is consistent with the modified plain information.

**Table 13:** NCCR and UACIT values of the encrypted information.

		DES	AES	Ref [36]	Ref [52]	Proposed
Text1	NCCR	54.55%	50.00%	9.30%	25.00%	99.48%
	UACIT	19.73%	16.92%	3.78%	2.26%	30.98%
Text2	NCCR	40.00%	20.00%	5.13%	12.50%	99.69%
	UACIT	11.50%	7.33%	2.01%	1.79%	32.93%
Text3	NCCR	39.14%	31.58%	5.00%	14.80%	99.84%
	UACIT	13.32%	12.25%	1.32%	1.16%	33.66%
Average	NCCR	44.56%	33.86%	6.48%	17.43%	99.67%
	UACIT	14.85%	12.17%	2.37%	1.74%	32.52%

As observed from Table 13, all the five encryption algorithms are sensitive to changes in plaintext information and all have a certain degree of resistance to differential attacks. By comparing the average values of NCCR and UACIT, it can be found that the lowest average value of NCCR is 6.48% for the encryption algorithm of literature [36] and the lowest average value of UACIT is 1.74% for the encryption algorithm of literature [52]. The average values of NCCR and UACIT of our proposed DEP encryption algorithm are 99.67% and 32.52%, respectively, and both UACIT and NCCR have been significantly improved. Consequently, fewer characters differed after encryption using the AES based on DNA algorithm, while the average degree of variation was lower after encryption using the algorithms based on DNA and hyperchaotic systems. Furthermore, both methods are weak against differential attacks and less sensitive to changes in plaintext information.

When a limited number of plaintext characters are changed, our proposed DEP algorithm has the feature that the key changes dynamically with the change of plaintext, in other words, the algorithm has the advantage of ’one-time-one-secret’. Moreover, when a few characters are changed, the key generated by the hash function can be very different, and the key obtained by the hash key decomposition algorithm can also be very dissimilar for each subsystem. Therefore, DEP algorithm is more sensitive to plaintext information, which makes the differential attack more challenging and makes it more impossible for the attacker to infer the key information.

## 5 Conclusions

For the requirements of high confidentiality and strict ethical regulation of privacy data in stem cell bank, this article proposed a double encryption protection (DEP) algorithm for stem cell bank privacy data based on improved AES and chaotic encryption technology.

1. DEP algorithm selects the hash value of plain information as the key. Our displayed hash key decomposition algorithm can generate subkeys for each component of the system through three conversion methods: dynamic encoding, dynamic encoding and cyclic shift, and conversion calculation, in order to meet the actual demands for keys in each subsystem. It realizes the ’one-time-one-secret’ encryption system, expands the key space, improves the key sensitivity and the ability to resist differential attacks.
2. In the first level of encryption, we perform three steps of dynamic DNA coding, byte filling based on DNA coding, and dynamic DNA sequence encryption based on AES. The DEP algorithm eliminates the restriction on the length of plain information, improves encryption efficiency, as well as enables dynamic encryption of private data.
3. In the second level of encryption, we put forward the sequence conversion algorithm which enable to generate operation mode sequence. The sequence of operation modes determines the subsequent encoding, operation, diffusion and decoding methods. The randomness of the cipher information and the sensitivity of the key are raised to ensure the security of private data.
4. In purpose of better evaluating the ability of text information to resist differential attacks, the number of character change rate (NCCR) and the unified average change intensity of text (UACIT) are submitted.

The DEP algorithm meets the high confidentiality requirements of private data in stem cell bank and has good application prospects in other related fields.
